# Supplementary figures and images for: Surmising synchrony of sound and sight: Factors explaining variance of audiovisual integration in hurdling, tap dancing and drumming
Source: PLoS One. 2021 Jul 22;16(7):e0253130. doi: 10.1371/journal.pone.0253130 (PMC8298114; doi:10.1371/journal.pone.0253130)

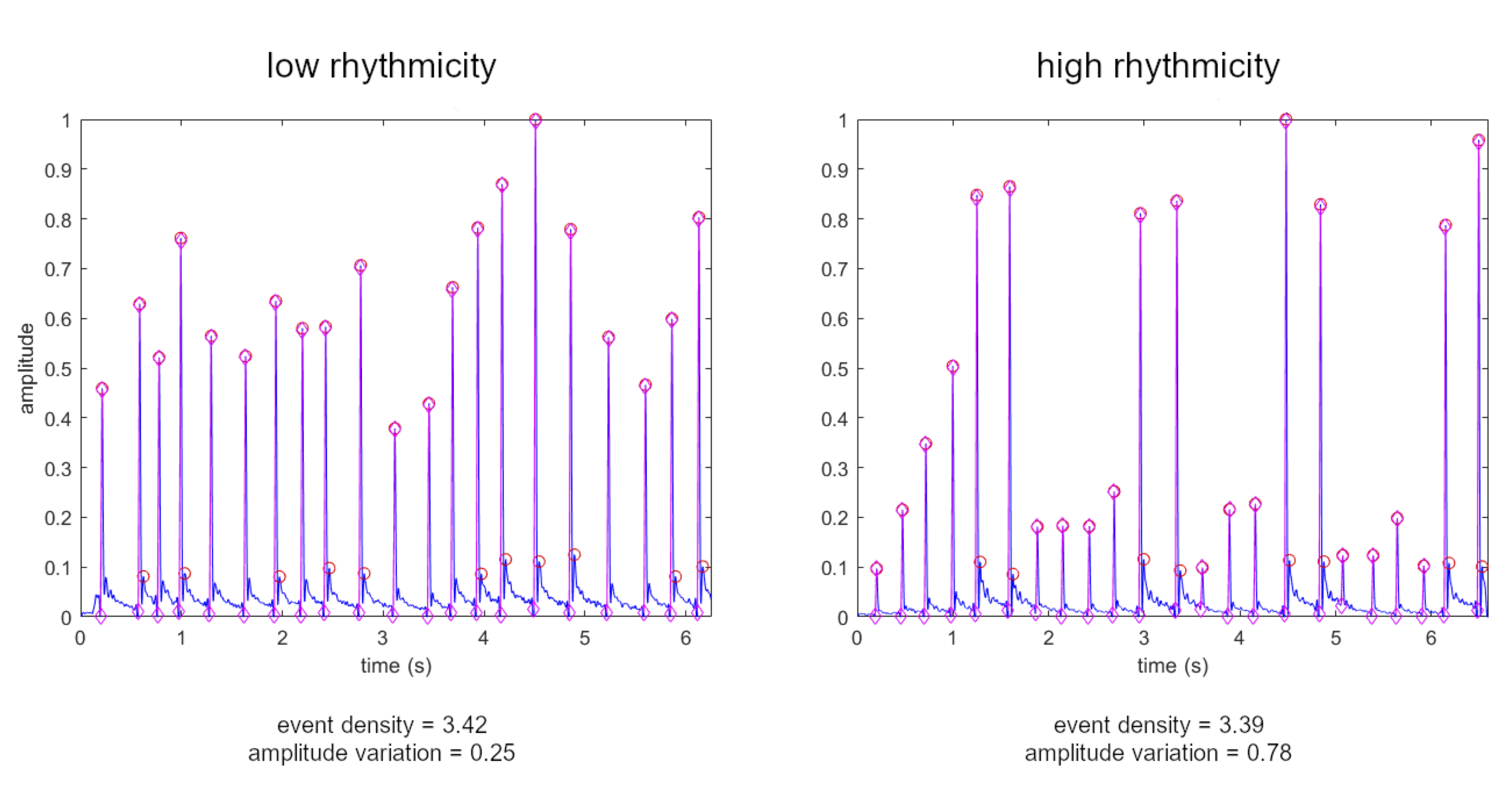

Supplement: S1 Fig — Rhythmicity was operationalized as variation of the amplitude envelope, shown here for two exemplary drumming sequences. While the event density of both recordings is virtually identical (3.42 and 3.39, respectively), the auditory events in the left recording are highly similar in loudness, resulting in low rhythmicity overall (v = 0.25). In contrast, the auditory events within the right recording vary more strongly in loudness, with almost equidistant duplets of loud (i.e. accentuated) events intersected with less accentuated events. This resulted in high rhythmicity overall (v = 0.78). (TIFF) [file pone.0253130.s001.tiff]

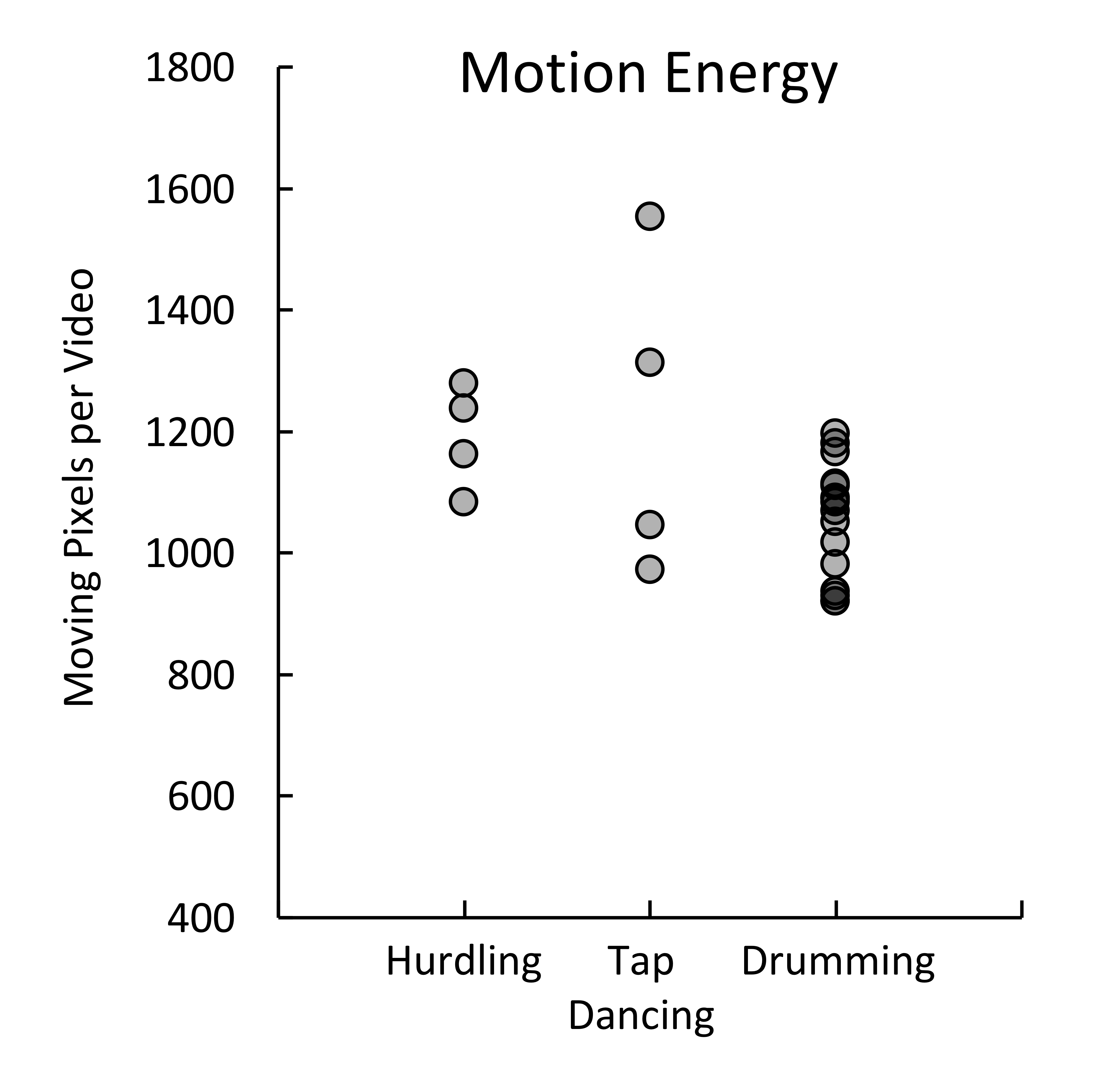

Supplement: S2 Fig — The amount of motion quantified by the amount of moving pixels per video for all PLD videos employed to generate different audio-visual asynchronous stimuli in Study 1 and Study 2. Each black marker depict the motion energy for one video (see Methods of Study 1 for details). (TIFF) [file pone.0253130.s002.tiff]
