# Supplementary material for: Surmising synchrony of sound and sight: Factors explaining variance of audiovisual integration in hurdling, tap dancing and drumming
Source: PLoS One. 2021 Jul 22;16(7):e0253130. doi: 10.1371/journal.pone.0253130 (PMC8298114; doi:10.1371/journal.pone.0253130)
Supplement: Attachment — Submitted filename: Response to Reviewers.docx [file pone.0253130.s016.docx]

**Reviewer #3**

The study addresses an interesting research question. Does perceived audio-visual synchrony depend on the intentionality of producing sounds? Two experiments are reported. In the first experiment tap dancing and hurdling point light videos are presented to participants at different audio-visual asynchronies. The authors observe a smaller window of perceived synchrony for the hurdling than the tap dancing actions. In order to assess the influence of event density and rhythm in their study, the authors then conduct a second experiment manipulating event density and rhythmicity using drumming stimuli and show that both factors significantly influence simultaneity judgements. Overall, the two experiments appear rather unrelated to each other and I am not convinced that the pattern of results is mainly driven by differences in task difficulty (simultaneity judgements are easier to make for hurdling than for drumming or tap dancing).

I have two major concerns related to the choice of experimental design and stimuli on the one hand and the lack of quantification of visual features of the actions on the other hand.

1) It does not become clear to me why the authors chose to compare tap dancing and hurdling in the first place, especially if the idea was to compare intentionally vs. accidentally produced action sounds. In order to show that intentionality matters, it would have been necessary to look at identical actions, and combining these with intentional or accidental action sounds. In the present experimental design intentionality is always confounded with the type of action being performed.

*We thank the reviewer for this important reference to the apparently still inadequate account of the motivation behind the choice of the types of movement studied. The current experiments built directly on a series of fMRI studies in which we investigated hurdling and tap dancing with respect to the perceptual processing of the sounds they produce. We added a new passage in the manuscript revision in the introduction and in the discussion, in which we hope to motivate the experimental approach more convincingly:*

*Introduction:*

*We decided to use two different types of sporting action that allowed us to study the processing of natural movement sounds in an ecologically valid context. This also had the particular advantage that the subjects' attention was not directed in any direction, since we created a completely natural perceptual situation.*

*Limitations:*

*Although it would have been possible to investigate audiovisual integration in the perception of intentionally and incidentally produced sounds using artificially generated stimuli, for the current series of experiments, the focus was on investigating natural movement sounds in an ecologically valid context. This also had the particular advantage of not biasing subjects' attention in any direction, since we were generating a quite natural perceptual situation. Another approach would have been to combine identical natural actions with intentional and incidental sounds. Here, however, we expected a confound in the sense that subjects would have expected the intentionally produced sound and not the incidental one. Thus, surprise or even irritation effects would have occurred in the incidental condition and would probably have strongly biased the comparison.*

Any observed effects can therefore be due to the fact that hurdling is different from tap dancing in many ways other than the intentionality of the action sounds. The second experiment addresses two auditory confounding factors (sound rhythm and event density), by introducing a third new action which is drumming. So the study leaves open many other ways in which these three actions differ both conceptually (artistic vs. competitive) and visually (with respect to their movement kinematics for example)

*This reference is also helpful for us because it gives us the opportunity to better motivate the choice of movements studied and to address possible limitations of our studies.*

*With regard to visual differences between the compared sports, we would like to point out here that our focus was on the topic of movement sounds. In our first revision, we added an analysis of visual kinetic energy according to the reviewer's instructions and found no significant differences here. In addition, we would like to point out that we used only point-light videos in both experiments and thus controlled for much of the visually irrelevant information. Please also consider furthermore our reply to point 2) raised by the Reviewer.*

*With regard to the conceptual differences raised by the reviewer, we agree that drumming as well as tap dancing are expressive and music-related actions whereas hurdling is not. Please note that we did not mean to directly compare drumming (Exp. 2) with hurdling or tap dancing (Exp. 1) as pointed out in the previous version of the manuscript: “Employing drumming PLDs enabled a direct control of event density and rhythmicity in an otherwise natural human motion stimulus. Note that using drumming actions, we kept intentionality of sound production constant while varying event density and rhythmicity as independent experimental factors. Since PLD markers were restricted to the upper body of the drummer, and since sounds were produced by handheld drumsticks in Study 2 as in contrast to sounds produced by feet in Study 2, we refrained from directly comparing conditions from Study 1 with Study 2.”*

*However, to more strongly acknowledge this point we now add another passage in the Discussion as follows:*

*While hurdling and tap dancing are comparable sports in many respects, they differ in terms of expressive or aesthetic appeal. Our studies reported here cannot rule out the influence of this factor, which should be the subject of future investigation.*

2) The rigorous assessment of auditory features is not matched by an equally rigorous assessment of the visual features of the stimuli, although such data appears to be available given that actions were recorded using motion capture.

The authors only report overall motion energy for the videos computed from the video. What about event density and rhythm in the visual domain? Could it be that synchrony during tap dancing and drumming are harder to detect because the movement amplitude of drumming and tap dancing is much smaller than that of hurdling? What about the saliency of cyclical motion or the influence of visual perspective? The difficulty of audiovisual integration does not only depend on saliency of auditory features but also of the visual stimuli, yet an assessment of how tap dancing, drumming and hurdling are different from each other with respect to their visual aspects and their movement kinematics is missing entirely.

*It is correct that the rigorous assessment of auditory features is not matched by an equally rigorous assessment of the visual features of the stimuli. While research on motion perception, not only in sports, has for many years focused heavily on and been limited to the visual domain, the focus of our research is clearly on how natural motion sounds are processed. We do not mean to deny the relevance of the visual domain, but to point out that the experiments we present relate specifically to motion sounds. In the first revision of our manuscript, in response to reviewer comments, we also included and added a descriptive and statistical analysis of visual stimulation. We point out this specific focus again in the revised discussion.*
